# Understanding the role of shame and its consequences in female hypersexual behaviours: A pilot study

**DOI:** 10.1556/JBA.3.2014.4.4

**Published:** 2014-12-18

**Authors:** MANPREET K. DHUFFAR, MARK D. GRIFFITHS

**Affiliations:** Nottingham Trent University, Nottingham, United Kingdom

**Keywords:** hypersexual behaviours, female sex addiction, shame, consequences

## Abstract

*Background and aims:* Hypersexuality and sexual addiction among females is a little understudied phenomenon. Shame is thought to be intrinsic to hypersexual behaviours, especially in women. Therefore, the aim of this study was to understand both hypersexual behaviours and consequences of hypersexual behaviours and their respective contributions to shame in a British sample of females (*n* = 102). *Methods:* Data were collected online via *Survey Monkey. Results:* Results showed the Sexual Behaviour History (SBH) and the Hypersexual Disorder Questionnaire (HDQ) had significant positive correlation with scores on the Shame Inventory. The results indicated that hypersexual behaviours were able to predict a small percentage of the variability in shame once sexual orientation (heterosexual vs. non-heterosexual) and religious beliefs (belief vs. no belief) were controlled for. Results also showed there was no evidence that religious affiliation and/or religious beliefs had an influence on the levels of hypersexuality and consequences of sexual behaviours as predictors of shame. *Conclusions:* While women in the UK are rapidly shifting to a feminist way of thinking with or without technology, hypersexual disorder may often be misdiagnosed and misunderstood because of the lack of understanding and how it is conceptualised. The implications of these findings are discussed.

## INTRODUCTION

Shame is thought to be intrinsic to hypersexual behaviours, especially in women (Gilliland, South, Carpenter & Hardy, 2011). Although many females may experience some temporary relief when engaging in the acting-out stage of Carnes’ (1983) cycle of sexual addiction, McDaniel and Valenti-Anderson (2012) noted that a significant number of women report that during and after the behaviour they feel shame, especially when they find themselves in the same situation of powerlessness repeatedly. This level of shame towards self confirms her negative core beliefs, the feeling of emotional discomfort and seeking ways to alleviate that distress which further perpetuates the cycle (Ferree, Hudson, Katehakis, McDaniel & Valenti-Anderson, 2012).

Additionally, the related review of the literature found gaps in our empirical knowledge of shame in female sex addicts. Firstly, studies into shame and hypersexuality have predominately been assessed in male samples (e.g., Reid, Harper & Anderson, 2009; Reid, Stein & Carpenter, 2011). Secondly, consequences of sexual behaviours are far greater for women than they are for men (i.e., STDs, infertility, unwanted pregnancies, legal issues such as prostitution, etc.) that lead to heightened shame. Thirdly, shame among female sex addicts often relies on theoretical speculations without empirical backing (e.g., the consideration of age as a predictor of levels of shame one may experience). Although shame has been described in various contexts that relates to sexual behaviour in females (i.e., societal disapproval [Wolfe, 2000]), it is often documented among women who are partners of sex addicts experiencing intensified levels of shame in an attempt to maintain secrecy from others (Schneider, 2000).

Previous literature in shame has reported that “shame disrupts the natural functioning of the self” (Kaufman, 1989, p. 5) and that it has an important social control function, but it can also distress. Therefore, if addiction is about the management of internal emotion, then shame is the primary feeling state that is medicated by addiction. Birchard (2004) states that the problem is that addiction – especially sexual addiction – creates a short-term fix of pleasure that is accompanied with high levels of shame. Carnes (1991) asserts that: “shame emerges from addiction. Shame causes addiction. Whichever way the shame is flowing, whether consequences or cause, it rests on one key personal assumption: somehow I am not measuring up” (p. 91).

A significant difference in women is that they are less likely than men to admit they are sexually addicted (Ferree et al., 2012). McDaniel and Valenti-Anderson (2012) noted that females will readily admit to their relationship dependence and acknowledge the use of seductive behaviours as – even after the sexual revolution – there is increased shame for a female to admit she is a sex addict. Paradoxically, it appears more shameful for a feminist to admit she is a love addict.

The relationship between consequences of sexual behaviour being a strong predictor of shame (e.g., Reid, 2010) has been demonstrated to be bi-directional, whereby shaming self-hostility could also be the strongest predictor of hyper-sexual behaviours (Reid et al., 2009). However, there appears to be a conundrum involved with the act of hyper-sexual behaviours and its consequences when observing shame, as there is a considerable overlap between both. For example, it has been suggested that clinical descriptions of treatment-seeking populations of hypersexual patients note intense feelings of guilt and shame (e.g., Reid, 2010), although, such feelings have been measured as a consequence of hypersexual behaviour (Garcia & Thibaut, 2010). Nevertheless, shame also seems to be positively associated with hypersexual behaviours.

Following the work of Reid (2010), Gilliland et al. (2011) observed the predicted connections between shame, guilt, and hypersexuality among a treatment-seeking sample. It was ascertained that the shame component had a significant, positive predictive relationship with hypersexual behaviours. While these findings do not establish a causal link between shame and hypersexual behaviours, they are, however, consistent with theories that hypersexual behaviours may be engaged as a maladaptive substitute of existing shame rather than viewing shame only as the result of such behaviour (Gilliland et al., 2011).

One factor that is often neglected in the shame literature is age. Weiss (2013b) suggests that age plays a significant role in which we now engage in sexual experiences. Due to the technological shifts in society, and the huge generation gaps between ‘digital natives’ and/or ‘screenagers’ (i.e., those individuals under 30 years) and ‘digital migrants’ (i.e., those individuals over 30 years), the level of shame experienced online is often reduced as a result of anonymity.

It should also be noted that, although the aforementioned have examined fundamental associations between the roles of shame in hypersexuality, they have largely neglected gender and age as a contributing factor in their analysis. Past findings have fuelled that the identification of such roles in females could potentially provide some empirical backing to support typical theoretical propositions as well as form treatment interventions that focus largely on diminishing levels of shame from the outset.

Given the literature outlined, the aim of this study was to extend previous findings by exploring and – to some extent – understanding both hypersexual behaviours and consequences of hypersexual behaviours (as distinct entities) and their respective contributions to shame in a British sample of females. It was hypothesised that, consistent with previous research (e.g., Gilliland et al., 2011; Reid et al., 2009), participants would indicate stronger feelings of shame pertaining to consequences of sexual behaviours and hypersexual behaviours (as indicated in the HDQ and HBI).

### Definition of hypersexual behaviour

As there remain unresolved questions about the addictive and compulsive conceptions of hypersexuality, and in line with others (i.e., Kafka, 2010; Reid, Stein et al., 2011), behaviour is defined in terms of its elements, consequences, and contexts, rather than in terms of presumed connections to other classes of conditions (i.e., such as having bipolar disorders). Although it was not considered in the DSM-5, the researchers’ conceptualisation of hypersexual behaviour is reflected in the classification of Hypersexual Disorder (Kafka, 2010).

## METHODS

### Participants and recruitment

The sample for this study was mainly recruited through the Internet. Respondents were sought from (a) *Twitter;* (b) *Facebook;* (c) postings on the website of the *Association for the Treatment of Sexual Addiction and Compulsivity* (ATSAC); and (d) poster advertising the study at a Mental Health clinic. The poster and recruitment statements said: “Female volunteers required for a research study in hyper-sexual behaviours. Please visit https://www.surveymonkey.com/s/GQT2SZS for more information”. Finally, participants were also recruited through personal contacts. Over a three-month period, 119 responses were collected. However, due to missing data, the final number of participants in this study comprised 102 females.

### Measures

Data were collected using a wide range of measures.

### Demographic information

Demographic data included questions about respondents’ city of residence, age, ethnicity, sexual orientation, and relationship status, level of education, employment, religion, religious beliefs and the degree to which the respondents had engaged in various sexual activities over the course of the past 12 months.

### Hypersexual Behaviour Consequences Scale (Reid, Garos & Fong, 2012)

The HBCS contains 21 items that concern various consequences encountered by hypersexual patients such as relationship problems, financial difficulties, job loss, sexual disease, diminished self-worth, and failure to keep important commitments. Each consequence is rated on a five-point scale (1 = *Unlikely to happen*, 2 = *Might happen*, 3 = *Will very likely happen*, 4 = *Has happened once or twice*, and 5 = *Has happened several times*, where higher scores reflect a greater presence and frequency of consequences. The scale has been used in college, community, and patient samples and has demonstrated high overall reliability (a = .95) and subscale reliability values of a = .91 on the Control subscale, a = .91 on the Coping subscale, and a = .89 on the Consequences subscale. Test–retest reliability in a sample of college students (*n* = 81) over a 2-week period was high for the total HBI score (*r* = .85), the Control subscale (*r* = .87), the Coping subscale (*r* = .87), and the Consequences subscale (*r* = .88). Confirmatory factor analysis has provided support for the factor structure, showing an acceptable goodness of fit with a root mean square error of approximation of .05 and a comparative fit index of .95 (Reid, Garos & Carpenter, 2011).

### Hypersexual Disorder Questionnaire (Reid, 2010)

Diagnostic criteria for HD have been adapted to a 10-item, self-report measure. Rather than simply assessing presence or absence of particular behaviours, the HDQ adopts a 5-point Likert response scale ranging from “Never” to “Almost Always” that allow for quantification of each symptom (e.g., I have been *unsuccessful* in my efforts to reduce or control the frequency of sexual fantasies, urges, and behaviours in my life; Reid, 2010). Responses were summed to yield a total score reflective of symptom intensity. The reliability coefficient for the measure shows high internal consistency (a= 0.95) among the items. A total HDQ score of 30 has been considered as the cut-off to imply problems with hypersexual behaviour; scores above that threshold (specific to this study) indicate that the respondent meets the criteria for hypersexual disorder.

### Hypersexual Behaviour Inventory – 19 (Reid, Garos et al., 2011)

The HBI is a 19-item self-report measure that assesses the degree to which respondents feel that their sexual thoughts, feelings, and behaviour are out of control (e.g., “My sexual behaviour controls my life”); the extent to which sexual behaviour is used as a way to cope with emotional discomfort (“Doing something sexual helps me cope with stress”); and the extent to which negative consequences are experienced due to their sexual activities (“My sexual thoughts and fantasies distract me from accomplishing important tasks”). Participants responded to each question on a 5-point Likert scale that ranges from 1 *(never)* to 5 *(very often)*. The overall reliability of the scale has shown to be high (a= .95), as have been the Control (a= .91), Coping (a= .91), and Consequences (a= .89) subscales. The HBI has demonstrated concurrent validity with the Compulsive Sexual Behavior Inventory (*r* = .92, *p* ì.01), the Sexual Compulsivity Scale (*r* = .82, *p* ì .01), and the Sexual Addiction Screening Test (*r* = .92, *p* ì .01). A total HBI score of 53 has been designated as the cut-off to signify problems with hypersexual behaviour; scores above that threshold indicate greater hypersexuality (Reid et al., 2009; Reid, Garos et al., 2011).

### Shame Inventory (Rizvi, 2010)

The Shame Inventory is a self-report measure designed to assess an individual’s propensity to experience shame both globally and in response to specific life events. The version for this study includes a definition of shame and three general items about the experience of shame. These questions ask about the frequency, intensity, and negative effects of shame each on a 5-point Likert scale, and were based on a similar measure designed to measure combat related guilt (Kubany, Abueg, Kilauano, Manke & Kaplan, 1997). These three items are followed by a list of 50 potential shame cues (i.e., events, behaviours, personal characteristics). An item pool for shame cues was generated by consulting with the literature on shame in addition to asking practicing clinicians to list a number of different situations that have elicited shame in their clients. The 50 final items were then selected on a rational basis. Participants were asked to rate each cue on a 0–4 scale to indicate the intensity of their current levels of shame about that event or characteristic, or to indicate if they have never experienced the event/behaviour/characteristic. The total score is the average rating on endorsed items and ranges from 0 to 4 with 4 indicating higher degrees of shame. The items show good internal consistency with an alpha coefficient of .80 and a test–retest reliability coefficient of .85 over a one-week time period. The SI inventory has also demonstrated convergent validity with two existing trait-based measures of shame and divergent validity with a measure of guilt. The SI has also successfully discriminated between clinical populations and healthy controls (Rizvi, 2010).

### Internet Related Activities (Daneback, Ross & Sven-Axel, 2006)

The Internet Related Activities measure was compiled by Daneback et al. (2006). The questions were adapted and modified for the purpose of this study (i.e., only 17 questions out of the 93 were utilised to measure Internet activities in the current sample). The self-reported measure was obtained from the first author who recommended that changes prior to distribution should be made as the questionnaire was devised in 2002. For this reason, three additional components were added: (i) Do you use social networking sites to find sexual partners? (ii) Do you feel that your cybersexual activities are out of control? and (iii) Are Internet-related activities having significant impacts on your daily life? The final measure consisted of 20 questions in total that were scored on a Likert scale.

### Procedure

The self-report measures existed as a web page created on *Survey Monkey* that captured the responses with one URL. The URL of the website was then distributed via popular social network sites (i.e., *Facebook, Twitter*) as well as on the *ATSAC* website. On each electronic posting, a link to the site of the questionnaire was provided along with a brief explanation of the study. Once participants had transferred to the site of the study, they were able to read the instructions, consent to the study, and complete the survey in their own time. Data were collected over a three-month period, while the URL was still available. All responses were collated, converted and sent to the researcher’s mailbox in format appropriate for *SPSS* analysis. Scores for each variable were cross-checked to ensure that no errors were made during the transference of responses into the database.

### Ethics

Ethical principles were carried out in accordance with the Declaration of Helsinki. The research team’s University Ethics Committee approved the study. All participants provided informed consent before participating in the study procedures.

## RESULTS

### Descriptive statistics

Mean age was not assessed as age was categorised into young adults (47.1% aged 18–29) and older adults (53.9% aged 30–42+). Ethnically, 87.3% were Caucasian. Participants indicated their marital status. Single participants represented 33.3% of the total sample, relationship (33.3%), married (29.4%), separated (2.9%), and widowed (1%). Education among the sample included college (3%), undergraduate (30.4%), postgraduate and higher (66.6%). Employment status was also reported, and 47.1% of the sample were students, 32.4% employed full time and 14.7% employed part-time. Sexual preference was mainly heterosexual (87.3%), although a few were homosexual (2%) and bisexual (10.8%).

Other demographic data relating to their general sexual activity included: frequency of vaginal intercourse, oral sex (give and receive), masturbation, telephone sex, cybersex, and pornography. Vaginal intercourse: less than six times a year (27.5%); once a month (19.6%); once a week (28.4%); and 2–4+ times a week (24.5%). Frequency of masturbation among the sample: less than six times a year (18.6%); once a month (29.4%); once a week (21.6%); and 2–4+ times a week (22.5%). Viewing of pornography: never (34.3%); less than six times a year (37.3%); once a month (22.5%); and weekly (5.9%). Two-thirds (65.7%) of the sample had viewed pornography at least once over the course of the past 12 months.

Table 1 displays the descriptive statistics for each scale including scale means, standard deviations, the potential range of scores for each scale and the alpha coefficients. As can be seen, reliability for all scales was satisfactory.

**Table 1.** Descriptive statistics including means *(M)*, standard deviations *(SD)*, range and Cronbach’s α

	*M*	*SD*	Range	α
Sexual Behaviour History	17.2	4.60	22	.68
Sexual Behaviour Consequences	29.6	10.87	47	.90
Hypersexual Disorder Questionnaire	18.3	5.93	28	.74
Hypersexual Disorder	31.7	10.86	43	.93
Inventory Shame Inventory	6.2	2.60	12	.94

### Correlations

Correlations among predictor and outcome variable(s) are presented in Table 2. This shows that shame was significantly correlated with only a few of the predictor variables (i.e., SBC and HBI). However, the only demographic variable that significantly correlated with shame was sexual orientation. Sexual Behaviour History (SBH) and the Hyper-sexual Disorder Questionnaire (HDQ) had a significant positive correlation with shame. Additionally, intercorrelation among predictor variables appeared to be high. Although some of the predictors were not statistically significant, they were entered into the regression model as exploratory variables.

**Table 2.** Pearson’s correlation and intercorrelations among variables concerning female sexual behaviour (*N* = 102)

Measure	1	2	3	4	5	6	7	8
1. Shame	–							
2. Age	–.016	–						
3. Sexual Orientation	–.229*	–.111	–					
4. Religious Belief	.118	.072	.044	–				
5. Sexual Behavior History	.190	–.150	.068	–.093	–			
6. Hypersexual Behavior Consequences	.391*	–.195	–.038	.000	.312**	–		
7. Hypersexual Disorder Questionnaire	.181	–.094	.191	–.090	.341**	.514**	–	
8. Hypersexual Behavior Inventory	.344*	–.128	.094	.067	.270*	.628**	.620**	–

*Note:* ** correlation significant at 0.01, * correlation significant at 0.05

### Regression model

A hierarchal multiple regression was employed to test variables that were the most significant predictors of shame. The results of the regression are reported in Table 3 (all insignificant variables were excluded from the table). In the model, the control variables were entered in the first step, followed by the main effect of shame in the second step.

**Table 3.** Unstandardised and standardised regression coefficients for the predictors of shame in female hypersexuality (*N* = 102)

		*b*	*SE b*	*b*
Step 1	Constant	3.773	1.416	
	Hypersexual Behavior Inventory	.076	.035	.308*
	Sexual Orientation (heterosexual vs. non-heterosexual)	–1.87	.868	–.243*
Step 2	Constant	3.522	1.374	
	Hypersexual Behavior Inventory	.033	.039	.133
	Hypersexual Behavior Consequences^†^	.084	.035	.359*

*Note: R =* .18 *for Step 1; R =* .25 *for Step 2 (ps <* .05). * p < .05. † Mediating variable

### Model summary

At Step 1 of the regression model, age, religious belief, sexual behaviour history and hypersexual behaviours (HBI and HDQ) collectively accounted for 10.9% of the variability in shame, *Adjusted R^2^ = .109, F*(6, 68) *=* 2.51*, p < *.05. At Step 2 of the model, the inclusion of consequences of sexual behaviour accounted for a total of 16.7% of variance in shame scores (in addition to the other variables). This incremental increase in *Adjusted R^2^* at Step 2 was statistically significant [*F*(1, 67) = 3.12, *p* < .05].

The regression also shows all six variables: sexual behaviour history, religious belief, age (younger vs. older), hypersexual behaviours (HBI and HDQ) significantly predicted shame (*R^2^* = .182 p < .05) in Step 1 of the model. However, hypersexual behaviours (only HBI scale) was the only variable that uniquely predicted shame, *b =* .08*, t(68) =* 2.16*, p ì *.05. In Step 2 of the model when all seven variables were combined (with the inclusion of SBC), all variables were significant predictors of shame (*R^2^* = .246 p ì .05). However, consequences of sexual behaviour emerged as the strongest variable that uniquely predicted shame, *b* = .08, *t*(67) = 2.39, *p* < .05.

### Model coefficients

Results from Step 1 of the model revealed that of six (controlled) variables, only hypersexual behaviours [HBI; *t*(68) = 2.16, *p* < .05] and sexual orientation [*t*(68) = –2.14, *p* < .05] were statistically significant predictors of shame. Step 2 showed that consequences of sexual behaviour (SBC) was the only variable that predicted shame, suggesting that it had the potential to mediate the association of hypersexual behaviours with shame.

### Mediation testing

**Figure 1.**
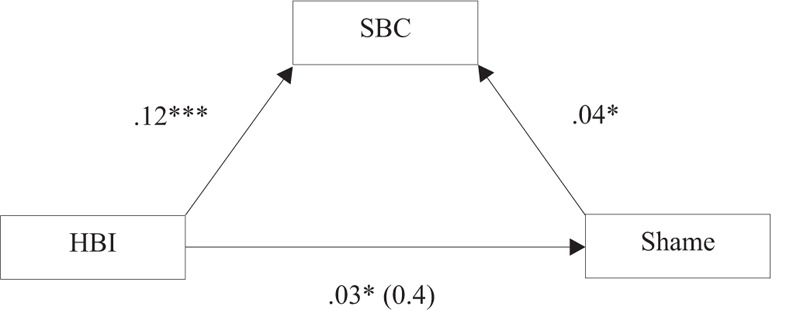
Testing sexual behaviour consequences (SBC) as a mediator variable of the association of hypersexual behaviours (HBI) with shame

*Note:* The coefficient in the parenthesis refers to the association between HBI and shame after SBC was introduced to the model (Sobel’s *z* = 2.04, *p* > .005). * *p* < .05, *** *p* < .0001

As shown in Figure 1, Baron and Kenny’s (1986) conditions for testing mediation were satisfied: The independent variable (HBI) significantly predicted the mediator (SBC) and the dependent variable (shame), and the mediator significantly predicted the dependent variable when the independent was controlled for. To test mediation, SBC was added to Step 2 of the regression model, resulting in a reduction in the coefficient of HBI from b = .45 (*p* < .001) to b = .13 (*p* = .397). The Sobel test was significant (*z* = 2.04, *p* < .005), indicating that the association of hypersexual behaviours (HBI) and shame were completely mediated by consequences of sexual behaviour.

### Age

To investigate the second research question, participants were categorised into two groups based on their age: (i) younger adults and (ii) older adults. An independent *t*-test was conducted to compare levels of shame for younger and older adults. There was a significant difference in scores for younger (*M* = 5.81, *SD* = 2.56) and older adults (*M* = 7.23, *SD* = 2.54); *t*(86) = –2.58, *p* = .011, two tailed). The mean difference was 1.42 with a moderate effect size (eta squared = .072).

## DISCUSSION

This study examined the role of shame in female hyper-sexual behaviours alongside consequences of such behaviours. The primary aim of investigation was to determine which variable(s) most significantly predicted shame. The results indicated that hypersexual behaviours (HBI and HDQ) were able to predict a small percentage of the variability in shame once sexual orientation (heterosexual vs. non-heterosexual) and religious belief (belief vs. no belief) were controlled for. Yet, while the full model regression significantly predicted shame, only consequences of sexual behaviours accounted for significant unique influence on shame and it is therefore in accordance with previous studies (e.g., Reid et al., 2009). Additionally, the association of hypersexual behaviours with shame was completely mediated by consequences of sexual behaviour (in Step 2 of the model).

As been previously reported (e.g., Reid, 2009), the relationship between consequences of sexual behaviour appears to be the strongest predictor of shame. Although a different measure of shame was employed in Reid’s study, it emerged that consequences of sexual behaviour and its relationship with shame were consistent among both males and females. It must also be noted that while findings may be similar, a distinction is that the sample in Reid’s study were men that had been diagnosed with hypersexual disorder whereas the current study was exploratory and comprised a non-clinical sample of females. While some females potentially met the criteria for hypersexual disorder (*n* = 6), a detailed assessment is warranted to ascertain the extent to which such behaviour is problematic.

Contrary to the hypothesis, there was no evidence to suggest that religious affiliation and/or religious beliefs had an influence on the levels of hypersexuality and consequences of sexual behaviours as predictors of shame. An explanation for this potentially may be that hypersexuality and/or sex addiction are umbrella terms for the particular forms of acting out (i.e., cybersex, pornography, masturbation and prostitution; Ferree, 2010) and the way in which they are undertaken. An activity such as cybersex perhaps may alleviate and/or diminish the levels of shame due to its anonymous nature. This, however, highlights that shame in traditional sex is significantly different to shame in modern (technology-driven) sex.

However, further tests revealed that there was a significant difference among younger and older females in the levels of shame experienced. One explanation for this could potentially be the marked changes in the experiences of sexual content and sexual contact in the past 25 years (i.e., access, affordability and anonymity; Delmonico, Griffin & Mori-arty, 2001). The way in which sexual materials and/or activities are accessed among a younger generation perhaps makes them less vulnerable to shame as they appear to be exploring their sexuality in anonymity and isolation, without the risk of being labelled. Weiss (2013b) describes that those who are under the age of 30 years are ‘digital natives’ and those above 30 years are ‘digital migrants’. More specifically, he argues that the prefrontal lobes of younger adults have evolved due to technology and that this has impacted on how young adults relate to others, mate, and work. Therefore, the neurobiological development and patterns of relat-edness in the younger generation are already different to their older counterparts, and what they perceive to be a relationship, dating and mating is synchronised to the electronic devices that they are introduced to from a relatively young age (Weiss, 2013a).

Although the HDQ scale was not a significant predictor of shame, it should be noted that the measure provided an indicator of how many females in the sample met the criteria for hypersexual disorder (missing values: *n* = 31). In the current sample 8.5% (6 participants out of 71) of respondents met the criteria for HD. Garcia and Thibaut (2010) suggest that:

Despite the lack of robust scientific data, a number of clinical elements, such as the frequent preoccupation with this type of behaviour, the time spent in sexual activities, the continuation of this behaviour despite its negative consequences, the repeated and unsuccessful efforts made to reduce the behaviour, are in favor of an addictive disorder. (p. 254)

### Limitations

This study was limited in several ways. First, this study was correlational, and therefore a causal direction among variables could not be established. The assumption was that hypersexual behaviours (including the HDQ) would be significant predictors of shame. Another major limitation of this study was in the self-selected sample and the modest number of participants, therefore the results should be interpreted with caution. As suggested by Azar (2000), those who volunteer to partake in an online study and/or experiment are self-selected. They are not a random sample of the population in general. This study utilised a convenient and self-selected group of social network users (where the research was promoted). Additionally, an online study also resulted in the distribution of a shorter questionnaire measuring shame, while the measure may have been completed in isolation and was anonymous, it must also be noted that being shameful is an aversive emotion in general and as sexual behaviours and consequences were measured alongside the shame inventory, participants may have responded in what they felt was socially desirable and may reflect a biased response. While the inventory provides a definition of shame prior to the participant completing the measure, it did not entirely measure the construct of shame in regards to specific contexts. Shame in this inventory enquired about the global feelings and the frequency to the extent that it occurred.

Promoting research on social network sites has the potential to generate a large sample size. However, due to the survey being online, it was not possible to determine whether or not some of the respondents had already been diagnosed with sexual addiction (since there is a probability that the six participants who met the criteria for sex addiction may have come from the *Association for the Treatment of Sexual Addiction and Compulsivity*). For this reason, further analysis could not be done. This study was also an exploratory study whereby a largely non-clinical sample was employed. While there is good internal consistency within the shame inventory, this study also successfully discriminates between clinical samples and healthy controls (Rizvi, 2010). In a clinical setting a low score of (for example) a depression scale does not necessarily highlight that the patient is not depressed, in fact, they can be disassociated and this too may be the case with some of the responses provided in the shame inventory.

The present results may have also been limited by the selection of measures. It is noteworthy that sexual behaviour consequences (SBC) completely mediated the association between the hypersexual behaviour inventory (HBI) and shame, suggesting that alternative measures may have produced stronger results. For example, the avoidance and withdrawal components of the Compass of Shame Scale (Elison, Lennon & Pulos, 2006) might have better reflected the relationship of shame with hypersexual behaviours. Rather than addressing shame directly, responses describe the feelings and behaviours. The TOSCA-3 (Test of Self Conscious Affect; Tangney, Dearing, Wagner & Gramzow, 2000) is another measure that has high reliability and validity among Western cultures and it measures both guilt and shame indirectly. However, it must also be reiterated that guilt and shame are often pooled together and the aims of this study were specifically to measure shame.

Other validated measures could potentially have been used to assess Internet sex activity such as the Internet Sex Addiction Screening Test (ISAST; Delmonico, 1997). Due to missing data, a number of variables that explored Internet-related activities were excluded from the final regression. Therefore, the study was unable to examine the relationship of cyber-related activities as a predictor of shame. The ISAST would have been a useful indicator of how many women were engaging in online activities and supported the influence of technology on age, rather than making inferences as to why there were distinctions between younger and older adults. Another limitation was that the hypersexual and consequences components were highly inter-correlated. Multi-collinearity among predictor variables can produce unstable regression coefficients, implying that these results should be replicated using alternative and less correlated measures.

### Future research

While this study is one of the few that has investigated the existence of female hypersexual behaviours and consequences accompanying such behaviours among a non-clinical British population, the results appear to provide empirical support for hypersexual disorder and its relationship with shame. Future investigations in measuring shame using qualitative methods, and further evaluations among those who meet the criteria for hypersexual disorder is warranted. Shame resulting from sexual behaviour could be assessed qualitatively by participants describing the impact of their sexual behaviour to self and how significant others may contribute to the shame they may experience.

Although there is good reliability and validity of the Hypersexual Disorder Questionnaire, a semi-structured interview may provide more insight in to the behaviours and consequences of sexually acting out, and how shame may accompany this. It must also be noted that sex in the digital age is not always physical, so the absence of derogatory labelling is something the younger adults may not experience unlike older adults that may have done prior to technology being readily available. In both cases, consequences and how they are experienced can potentially have an impact on how levels of shame may be interpreted. For example, younger adults may experience a delay in which the feeling of shame takes primacy (having cybersex and only feeling shameful when a consequence has occurred), whereas older adults may feel shame immediately and prior to a consequence even occurring. However, this is a speculation and does not necessarily reflect reality and there appears to be a paradox involved in both cases.

Based on the demographic information from the participants, it appeared that while the frequency of sexual behaviours may have been physical intercourse, over 65% of the sample had viewed pornography in the past year. Future research could also focus on the content that is viewed, as some pornographic material may be socially unacceptable, resulting to internal shame. Furthermore, a clinical and a healthy control group or treatment vs. no treatment comparisons could also generate a different set of results, whereby the researcher is present to collect baseline and follow up measures from both sets of groups.

There are several other findings from this study that potentially prompt future research, it may be worthwhile to: (i) conduct studies in person to avoid missing data; (ii) investigate whether cyber-related sexual behaviours are predictors of shame but splitting the sample into three categories of age (i.e., under 25, 25–29 and 30+ years) to observe in depth how age influences the levels of shame with respect to hypersexual behaviours and consequences; (iii) investigate cultural differences and how they may influence levels of shame despite residing in a western society; and (iv) investigate culture being viewed as a potential contributing factor to the onset of Internet sex addiction among females who are among strict religious families. There is a significant need for shame and consequence led outcome studies among hypersexual women to identify the types of protective factors needed and awareness of the extent to which such behaviours can be harmful.

## CONCLUSIONS

It is hoped that the applicability of these exploratory findings can extend to a clinical population within the U.K and can be incorporated into gender-specific treatments with a greater understanding of the harmful consequences of sexual behaviour as well as the shame that accompanies it. While women in the UK are rapidly shifting to a feminist way of thinking with or without technology, hypersexual disorder may often be misdiagnosed and misunderstood because of the lack of understanding we have on how it is conceptualised.
